# Abdominal Section in Perforating-Ulcer of the Stomach. A Suggestion

**Published:** 1883-12

**Authors:** Nelson C. Dobson

**Affiliations:** Surgeon to the Bristol General Hospital; and Lecturer on Surgery in the Bristol Medical School


### ABDOMINAL SECTION IN PERFORATING-ULCER OF THE STOMACH.

### A SUGGESTION.

BY

NELSON C. DOBSON, F.R.C.S.,

*Surgeon to the Bristol General Hospital; and Lecturer on Surgery in the Bristol Medical School.*

---

A CASE of perforating-ulcer of the stomach, which terminated fatally in sixteen hours from the beginning of the symptoms, suggested to me the possibility of successfully attempting some surgical interference in similar cases.

The brief history of this particular case is as follows: —I was called at 9.30 p.m. to see a small, wiry, energetic woman, age 27, the parlour-maid to one of my patients. I was told that up to within three hours of my visit she had been perfectly well, in fact she had over and above her ordinary duties walked four or five miles. About six o'clock in the evening she first complained of feeling ill, she was then more or less faint, and had pain in her stomach. When I saw her I expressed the opinion that she was gravely ill and suffering from something abdominal, either a perforation of the stomach from an old ulcer, or some hæmorrhage into the abdominal cavity. I ordered her what I considered suitable treatment, and saw her very early the next morning; she was then much worse, and died in a few hours. When I first saw her she was pale and faint, lying on her right side, with blanched lips, a pulse of 116 of fair power; she had pain at the epigastrium, tenderness more or less over the abdomen; but she chiefly complained of pain above the left clavicle. She had no vomiting, but there had been slight attempts at retching; subsequently the ordinary symptoms of peritonitis developed themselves.

I was led to think she had either perforation of the stomach or pelvic hæmatocele (she had menstruated a few days previously).

*Post-mortem.*—On opening the peritoneal cavity I found lying over the front surface of the stomach a considerable quantity (at least a pint) of fluid, evidently stomach-contents, though there was but little solid material; the fluid did not appear to have diffused itself very widely over the general peritoneal cavity; there was evidence of lymph here and there, and on lifting up the left lobe of the liver, which was slightly adherent to the front wall of the stomach, a small hole was discovered in the anterior surface of the latter organ midway between the pylorus and the cardia, and about one inch from the lesser curvature.

These are the facts of the case, and in themselves they present nothing unusual; but it appeared to me, especially by the light of the post-mortem appearances, that if one had had the courage of one's convictions some attempt might have been made to rescue the patient by surgical means.

It was, as was shown by the post-mortem, a perforation of the stomach in a very accessible situation, and the extravasated stomach-contents, which were chiefly fluid, were readily within reach of the cleaning-up process resorted to in most cases of abdominal section, and which my own practical experience in ovariotomy assures me might have been successfully accomplished in this case.

The actual practice of abdominal surgery has made rapid advances during the past few years; and the suggestive papers of Marion Sims and others on gun-shot wounds of the abdomen and rupture of the bladder, &c., have still further opened the field of possibilities in abdominal injuries and diseases; but, so far as I know, no suggestion has yet been made to treat surgically such a condition as was illustrated by the case I have mentioned.

It is true that Billroth has successfully removed the pylorus and has even suggested the idea of removing simple ulcers of the stomach; a suggestion which is much more daring, and, to my mind, by no means so justifiable as the one which I venture to make.

I know that my suggestion with reference to surgical interference will be discouraged by the fact that the difficulties of exact diagnosis are great; but these difficulties beset us in all abdominal cases in which we are now accustomed to operate. If we once recognize the possibility of surgical interference in perforating-ulcer of the stomach it will stimulate us to increased accuracy of diagnosis, and I do not despair that before long we shall have in these cases, as we already have in many other abdominal disorders, sufficiently rational and sound data to guide us; if, on the other hand, we are content to accept Leube's opinion "that when perforation occurs—that most disastrous event in the course of gastric ulcer—the only treatment in most cases is to induce euthanasia," then we shall probably rest where we are; but my hope and belief is that before long this disorder, which is so commonly fatal, will be robbed of some of its perils by the timely and well-directed efforts of the surgeon.

It occurred to me that in such a case as mine serviceable interference might assume various forms.

1st. Simple abdominal section with cleaning-up of peritoneum, leaving the gastric ulcer to heal of itself, with appropriate feeding by the rectum for a time.

2nd. In addition to the above means, by paring the edges of the ulcer, or by bringing the edges of the ulcer together without paring, inserting the stitches after the manner employed by Billroth in his pyloric resections.

3rd. Stitching the ulcer to the abdominal wall and establishing a gastric fistula.

Of course, I am aware that all perforating-ulcers of the stomach do not occur on the anterior surface of that organ, and therefore that some modification of the plans proposed would have to be adopted in such cases. The tearing through of a large portion of the great omentum, or the bodily lifting up of this structure, so as to inspect and do what might be needful to the posterior wall of the stomach, need not deter the operator, for it has been proved by experience that this may be done without fatal issue.

Another instance (though not one of perforating-ulcer) occurs to me in which I now think abdominal section might have been satisfactorily practised. It was a case of ruptured tubal gestation, which terminated fatally, with hæmorrhage into the pelvis. This case was accurately diagnosed during life by the late Dr. Martyn. It occurred some years ago, before abdominal surgery was as well understood as it is now; I have no doubt similar cases might be and perhaps are now treated by surgical means.

I put forward the suggestion contained in this paper without waiting for an opportunity of carrying out that suggestion, because it is probable that it may be a long time before another similar case will be met with by me, though they will be certain to occur in the practice of others, who may bear in mind my suggestion and possibly act upon it.

